# Identification of Putative Biosynthetic Gene Clusters for Tolyporphins in Multiple Filamentous Cyanobacteria

**DOI:** 10.3390/life11080758

**Published:** 2021-07-28

**Authors:** Xiaohe Jin, Yunlong Zhang, Ran Zhang, Kathy-Uyen Nguyen, Jonathan S. Lindsey, Eric S. Miller

**Affiliations:** 1 Department of Chemistry, North Carolina State University, Raleigh, NC 27695-8204, USA; xjin4@ncsu.edu (X.J.); yzhang95@ncsu.edu (Y.Z.); rzhang12@ncsu.edu (R.Z.); uvnguyen@ncsu.edu (K.-U.N.); 2 Department of Plant and Microbial Biology, North Carolina State University, Raleigh, NC 27695-7615, USA

**Keywords:** tolyporphins, tetrapyrroles, cyanobacteria, biosynthetic gene cluster, *Brasilonema*, *Nostoc*, *Oculatella*

## Abstract

Tolyporphins A–R are unusual tetrapyrrole macrocycles produced by the non-axenic filamentous cyanobacterium HT-58-2. A putative biosynthetic gene cluster for biosynthesis of tolyporphins (here termed BGC-1) was previously identified in the genome of HT-58-2. Here, homology searching of BGC-1 in HT-58-2 led to identification of similar BGCs in seven other filamentous cyanobacteria, including strains *Nostoc* sp. 106C, *Nostoc* sp. RF31YmG, *Nostoc* sp. FACHB-892, *Brasilonema octagenarum* UFV-OR1, *Brasilonema octagenarum* UFV-E1, *Brasilonema sennae* CENA114 and *Oculatella* sp. LEGE 06141, suggesting their potential for tolyporphins production. A similar gene cluster (BGC-2) also was identified unexpectedly in HT-58-2. Tolyporphins BGCs were not identified in unicellular cyanobacteria. Phylogenetic analysis based on 16S rRNA and a common component of the BGCs, TolD, points to a close evolutionary history between each strain and their respective tolyporphins BGC. Though identified with putative tolyporphins BGCs, examination of pigments extracted from three cyanobacteria has not revealed the presence of tolyporphins. Overall, the identification of BGCs and potential producers of tolyporphins presents a collection of candidate cyanobacteria for genetic and biochemical analysis pertaining to these unusual tetrapyrrole macrocycles.

## 1. Introduction

Cyanobacteria inhabit diverse environments and contain rich biosynthetic capacity for formation of diverse molecular structures [1,2,3,4,5,6,7]. In 1992, a team at the University of Hawaii reported a novel natural product in a lipophilic extract from the culture containing the cyanobacterium *Tolypothrix nodosa* [8]. The agent, a glycosylated tetrapyrrole macrocycle termed tolyporphin A, reversed multidrug resistance in SKVLB1 carcinoma cells in vitro [9]. The cyanobacterial culture, labeled HT-58-2, was collected on the island of Pohnpei in Micronesia. Since then, a family of tolyporphin analogues (B–R) has been identified in the HT-58-2 culture [10,11,12]. Tolyporphins are the only new class of tetrapyrrole macrocycles reported since the discovery of the F_430_ coenzyme of methanogenesis.

**Figure 1:**
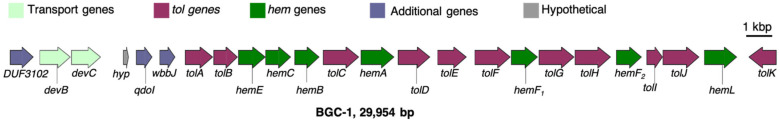
The putative tolyporphins biosynthetic gene cluster (BGC-1) in the HT-58-2 cyanobacterial genome [13]. BGC-1 contains tetrapyrrole biosynthesis genes (*hem*) that are also found dispersed throughout the genome of HT-58-2, and other genes (*tol*) predicted to be involved in the biosynthesis of tolyporphins.

The HT-58-2 culture is non-axenic, comprised of a single filamentous cyanobacterium and a collection of community bacteria, including those attached to the sheath of the cyanobacterial filaments [13,14]. Growth under conditions of environmental stress (e.g., deprivation of soluble nitrogen) over the period 4–7 weeks elicits production of tolyporphins, which can accumulate to a level that rivals the quantity of chlorophyll [15]. The tolyporphins preferentially localize in the membrane sheath of the cyanobacterium [16]. The chromosome of the cyanobacterium is circular and composed of 7.85 Mbp [13]. Tetrapyrrole-related genes are generally distributed throughout the genome [13]. On the other hand, a biosynthetic gene cluster (here termed BGC-1) containing tetrapyrrole-related (*hem*) genes and other genes (here termed *tol*) was identified and proposed for the biosynthesis of tolyporphins (Figure 1) [13]. Recent analysis has revealed several putative biosynthetic gene clusters of other natural products including tolypodiols, hapalosin, anatoxins, shinorine, and heterocyst glycolipids [17]. These findings have established a framework for the investigation of tolyporphins biosynthesis and the regulation thereof in the HT-58-2 cyanobacterium-bacterial consortium. Yet, to date the HT-58-2 culture remains the only known producer of tolyporphins.

The identification of BGC-1 prompted consideration whether similar BGCs might be present in other cyanobacteria. The worldwide inventory of cyanobacteria is large, composed of culture collections (estimated > 1000 samples) as well as complete genome sequences (~400 in Cyanobase). A cursory search of Genbank, which contains publicly available genomic information across organisms, did not reveal the presence of other analogous BGCs. A manual search of Genbank was carried out to identify other organisms that contain the clustered genes found in BGC-1. This paper presents a bioinformatic search of potential tolyporphins producers using homology with BGC-1 as a guide, which has identified similar BGCs in seven additional filamentous cyanobacteria, and unexpectedly, a second BGC (BGC-2) in HT-58-2. The work provides a deeper genomic foundation for probing tolyporphins biosynthesis.

## 2. Materials and Methods

### 2.1. Identification of Tolyporphins BGCs Tol Functions and BGCs from Other Cyanobacteria

The amino acid sequences of proteins encoded by the putative *tol* genes in BGC-1 from HT-58-2 were set as queries to perform BLASTP similarity searching against the non-redundant protein sequence database [18]. Genomes and genomic contigs containing corresponding Tol-like proteins were further investigated manually to ascertain whether such proteins were arranged in gene clusters. The Genbank accessions of genomic DNA identified as containing BGCs with multiple *tol* and *hem* genes are as follows: cyanobacterium sp. HT-58-2 (CP019636), *Nostoc* sp.106C (MTAW01000098), *Nostoc* sp. RF31YmG (MTAX01000063), *Nostoc* sp. FACHB-892 (JACJTR010000005), *Brasilonema octagenarum* UFV-OR1 (QMEC01000008 and QMEC01000114), *Brasilonema octagenarum* UFV-E1 (CP030121), *Brasilonema sennae* CENA114 (CP030118) and *Oculatella* sp. LEGE 06141 (JADEWO010000054).

### 2.2. Phylogenetic Analysis

The 16S ribosomal RNA sequences from the selected cyanobacteria were first aligned with ClustalW using default settings in MEGA X. Following removal of end gaps, the aligned sequences were used in phylogenetic tree construction using neighbor-joining (NJ) methods [19] in MEGA X [20,21]. Statistical confidence of the inferred evolutionary relationships was assessed by bootstrapping (1000 replicates) [22]. The evolutionary distances were computed using the Maximum Composite Likelihood method [23] and are in units of base substitutions per site. For evolutionary analysis of protein sequences, proteins were aligned with ClustalW, followed by end gap deletion, and the inferred phylogenetic tree obtained by the neighbor-joining method [19]. Evolutionary distances were computed using the Poisson correction method [24].

The accession numbers for 16S rRNA analysis are as follows: *B*. *octagenarum* UFV-E1 (EF150854), *B*. *octagenarum* UFV-OR1 (EF150855), *B*. *sennae* CENA114 (EF117246), *Oculatella* sp. LEGE 06141 (KU951789), *Nostoc* sp. PCC 7120 (X59559), *B*. *bromeliae* SPC 951 (DQ486055), *Nostoc* sp. FACHB-892 (KF494241), and *Tolypothrix* sp. NIES-4075 (LC497426). 16S rRNA sequences were extracted from the genome entries for *Nostoc* sp. 106C, *Nostoc* sp. RF31YmG, *Scytonema* sp. NIES-4073, *Calothrix* sp. NIES-4071, and cyanobacterium HT-58-2. All 16S rRNA sequences were trimmed to the same length of 1027 bp.

Accession numbers for TolD-type protein sequences are as follows: cyanobacterium HT-58-2 (ARV58988), *B*. *octagenarum* UFV-E1 (QDL13739), *B*. *octagenarum* UFV-OR1 (NMF65696), *B*. *sennae* CENA114 (QDL07377), *Oculatella.* sp. LEGE 06141 (MBE9181921), *Nostoc.* sp. 106C (OUL31080), *Nostoc.* sp. RF31YmG (OUL25682), and *Nostoc.* sp. FACHB-892 (MBD2726601).

### 2.3. Cyanobacterial Strains—Identification and Procurement

Seven organisms were identified by BLASTP similarity searching against the non-redundant protein sequence database Genbank, as described in Section 2.1. Of the seven examined with regards to genomics, three were obtained and studied in our laboratory. The *Nostoc* sp. 106C sample was provided by Dr. Francisco (Paco) Barona-Gómez and Dr. Angélica Cibrian Jaramillo. The *Nostoc* sp. RF31YmG sample was not available. The *Nostoc* sp. FACHB-892 sample was collected in a protected region of China and hence not sought by us. The *B. octagenarum* UFV-E1 sample was provided by Dr. Marcelo Gomes Marçal Vieira and Dr. Diego Genuário. Inquiries to procure *B*. *octagenarum* UFV-OR1 and *B*. *sennae* CENA114 were not successful. The *Oculatella* sp. LEGE 06141 sample was provided by Dr. Pedro Leão. Thus, three of seven strains were obtained for culture studies.

### 2.4. Examination of Extracts from Cyanobacteria for Tolyporphins

The three samples (*Nostoc* sp. 106C, *B*. *octagenarum* UFV-E1, and *Oculatella* sp. LEGE 06141) were incubated in BG-11 medium as described previously [13] under continuous white light (62 µmol m^−2^ s^−1^) at 28 °C with shaking at 120 rpm for 25 days. HT-58-2 was grown identically for 30 days. The four samples were also incubated in BG-11o medium [13] containing NH_4_HCO_3_ (1.87 mM) for 25 days before collection for pigment extraction. A 2-mL sample of each culture was collected and washed twice with phosphate-buffered saline. The pellets were vigorously extracted three times using a Mini-beadbeater (BioSpec) with 1 mL of CH_2_Cl_2_/2-propanol (*v/v* = 1/1), and centrifuged at 13,000× *g* for 3 min at 4 °C. The resulting supernatants containing extracted pigments were combined and subjected to analysis by absorption spectroscopy [15], by HPLC with absorption spectroscopic detection [15], and by a fluorescence assay [25] following the specific established protocols, which have been described at great length [15,25].

## 3. Results

### 3.1. Significant Gene Features in BGC-1

**Figure 2:**
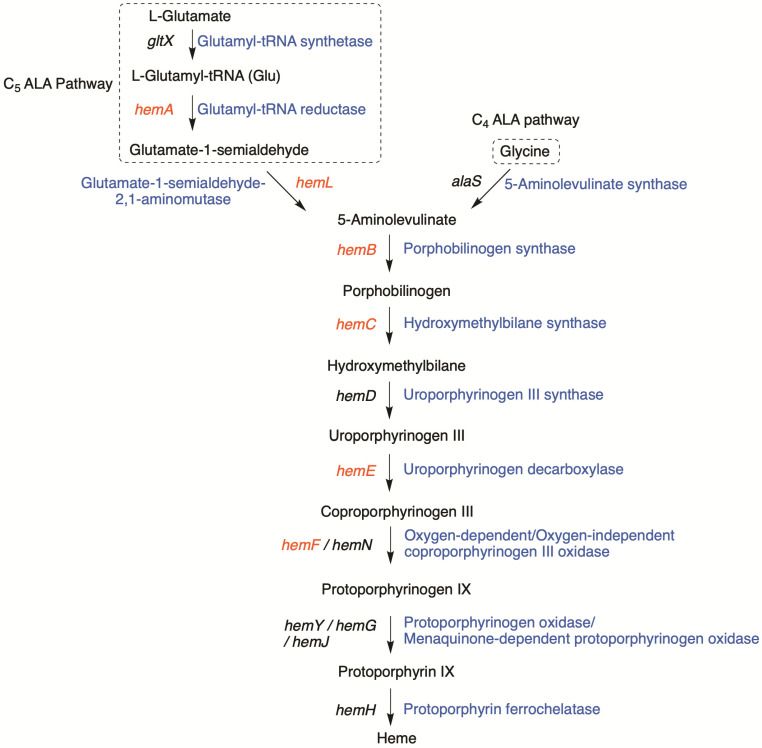
The core pathway of tetrapyrrole biosynthesis in prokaryotes beginning with L-glutamate (as in HT-58-2) or L-glycine. The classic ‘*hemX*’ nomenclature describes genes encoding enzymes in the tetrapyrrole pathway, where the encoded enzymes are displayed in blue. The *hem* genes that are present in BGC-1 are highlighted in red.

**Figure 3:**
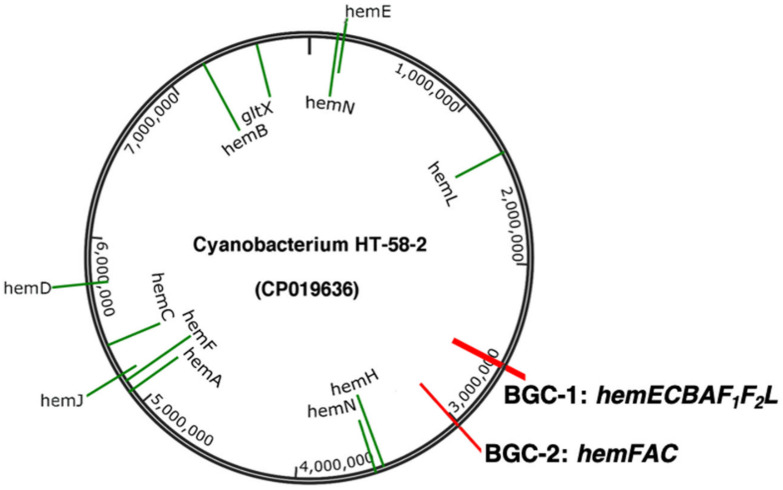
Distribution of *hem* genes throughout the genome of cyanobacterium HT-58-2 (7.85 Mbp), except in the two proposed tolyporphins clusters (BGC-1 and BGC-2).

The core pathway for the biosynthesis of tetrapyrroles is derived from two distinct precursors (C_5_ glutamate and C_4_ glycine) to 5-aminolevulinic acid (ALA). In three conserved reactions, ALA is converted to uroporphyrinogen III that serves as the universal branch-point for tetrapyrrole biosynthesis (Figure 2) [26,27]. Genes encoding enzymes in the core pathway of tetrapyrroles are known as *hem* genes. In BGC-1, seven clustered *hem* genes (*hemABCEF_1_F_2_*) are observed, coding almost all enzymes of the core pathway from L-glutamyl-tRNA (Glu) to protoporphyrinogen IX, as shown in Figure 1. The two exceptions are *gltX* and *hemD*, which encode glutamyl tRNA synthetase and uroporphyrinogen synthase, respectively. Each of the seven *hem* genes in BGC-1 also appears at least once distributed throughout the HT-58-2 genome (Figure 3).

The clustering of *hem* genes is known in Gram-positive bacteria [28,29,30]. On the other hand, to our knowledge, *hem* genes tend to be dispersed in Gram-negative bacteria, as is the case with *Escherichia coli* [31,32]. In addition to *hem* genes, eleven additional genes, termed as *tol* genes herein, are assigned provisionally on the basis of homology analysis and are considered likely to code for enzymes that participate in specific steps of the biosynthesis of tolyporphins. While gene assignments and predicted functions will be described in depth elsewhere, for purposes of presentation here, the provisional assignments for the selected *tol* genes (encoded proteins) are as follows: *tolA* (dTDP-glucose 4,6-dehydratase), *tolB* (glucose-1-phosphate thymidylyltransferase), *tolC* (acyltransferase), *tolD* (glycosyltransferase), *tolE* (UDP-glucose 4-epimerase), *tolF* (aminotransferase), *tolG* and *tolH* (cytochrome P450), *tolI* (L-2-amino-thiazoline-4-carboxylic acid hydrolase), *tolJ* (FAD-binding protein), and *tolK* (aldo/keto reductase). Another feature of the BGC-1 (left-most region) is the presence of genes for secretory and transport proteins. Above all, the uncommonly clustered *hem* genes suggest a significant marker for recognizing presumptive BGCs in other possible producers of tolyporphins.

### 3.2. Homology Searching for Additional Tolyporphins BGCs

**Figure 4:**
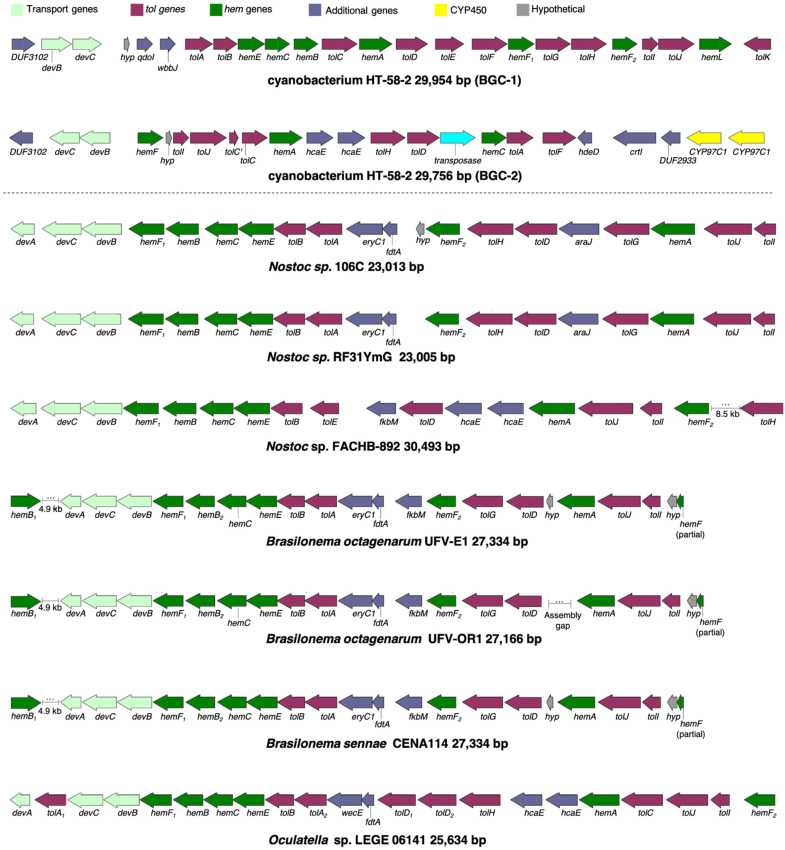
Tolyporphins BGCs identified in filamentous cyanobacteria. BGC-1 from HT-58-2 is compared to HT-58-2 BGC-2 and seven newly detected clusters (each from distinct cyanobacteria). Core tetrapyrrole biosynthetic genes (*hem*) are green, *tol* genes are maroon, adjacent transport genes are light green, cytochrome P450s are yellow, other identified genes are dark blue, and unknown function genes are gray. Genbank accessions for the map data are in Table S1.

To pursue other BGCs for tolyporphins that resemble BGC-1 in strain HT-58-2, further analysis of genomic data of the HT-58-2 cyanobacterium and comparative genomics of other filamentous cyanobacteria were conducted using the sequence of BGC-1 as a query (Figure 1) [13]. We used manual BLASTP database searching, rather than more automated methods similar to AntiSMASH [33], MultiGeneBlast [34], and cblaster [35] since the definitive role of tolyporphins BGCs has not been established, nor has the HT-58-2 BGC-1 been incorporated into commonly utilized BGC search engines. While our focus was to look outward to other organisms, we unexpectedly found a second, less extensive putative tolyporphins BGC (termed BGC-2) in HT-58-2 at region 2,994,941–3,043,548 bp. This 29.7 kbp cluster contains three *hem* genes and eight *tol*-like genes, in contrast to the six *hem* genes and eleven *tol* genes in BGC-1. The alignment between BGC-1 and BGC-2 is shown in Figure 4.

In BGC-2, seven proteins are aligned with over 50% identity to Tol proteins from BGC-1 (TolACDHIJ), and are all arranged in the same orientation (+). Two TolC-like proteins (TolC’ and TolC) are present in BGC-2. TolC’ and TolC align with TolC at N- and C-regions, respectively, which may suggest a frameshift or other DNA sequencing error. Unlike BGC-1, there is only one cytochrome P450 in BGC-2, sharing higher identity to TolH (CYP88A). However, two other P450 genes (yellow arrows in Figure 4) are adjacent to the cluster. Additionally, duplicate *hcaE* genes encoding aromatic ring-hydroxylating dioxygenases [36] are present within the BGC-2 region. The relevance of these additional genes in BGC-2 to tolyporphins biosynthesis is unknown. Similar to BGC-1, three transport-related protein genes (DUF3102 domain-containing proteins DevB and DevC) are present at the left-most end of BGC-2. Further studies are required to identify the roles of BGC-1 and BGC-2 in the biosynthesis of tolyporphins.

In addition, seven cyanobacterial genomes were identified with similar *hem* and *tol* genes, including *Nostoc* sp.106C, *Nostoc* sp. RF31YmG, *Nostoc* sp. FACHB-892, three *Brasilonema* isolates, and *Oculatella* sp. LEGE 06141 (Table 1). The alignment of all BGCs is shown in Figure 4.

Additional information concerning each of the seven strains is as follows:

- The genomic sequences of *Nostoc* sp.106C and *Nostoc* sp. RF31YmG were obtained from endophytic sub-communities from coralloid roots of *Dioon merolae* followed by metagenomic sequencing [5]. These *Nostoc* spp. cultures are nonaxenic, as is the case for the HT-58-2 culture. Indeed, both contain gene clusters of ~23 kbp that align with the tolyporphins BGC-1 in HT-58-2 (Figure 4). BLASTP alignments revealed that most Tol-like proteins from the two *Nostoc* strains align with relatively high identity to those from HT-58-2 BGC-1 (Table S1).
- *Nostoc* sp. FACHB 892 was obtained from soil crusts in the Tengger Desert, China, for extracellular polysaccharide studies [37]. The total length of the BGC is ~30 kbp with an extended 8.5 kbp to include the nearby *tolH* gene.
- The three *Brasilonema* strains were isolated and described from Brazil [38], wherein *hem-tol* clusters of ~27 kbp were observed in all three strains (Figure 4).
- *Oculatella* sp. LEGE 06141 is from the Blue Biotechnology and Ecotoxicology Culture Collection in Portugal, where many of the LEGE strains are non-axenic [39].

Strain LEGE 06141 contains duplicate *hcaE* genes as observed in HT-58-2 BGC-2 and the BGC of *Nostoc* sp. FACHB-892. Proteins similar to five *tol* gene products (TolABDIJ) are identified in all predicted BGCs from the cyanobacteria (only *tolA* is absent in *Nostoc* sp. FACHB-892), and at least one of the cytochrome P450s (TolG or TolH) is always present. None of the newly identified BGCs encode the TolF aminotransferase or TolK aldo/keto reductase (although similar proteins are encoded elsewhere in the genomes). The remaining *tol* genes are variably present in certain strains, i.e., *tolC* (acyltransferase) in *Oculatella* sp. LEGE 06141 and *tolE* in *Nostoc* sp. FACHB-892. A summary of similarities among HT-58-2 tolyporphins BGC-1, BGC-2, and all other BGCs is provided in Table S1. A partial BGC containing two *hem* and five *tol* genes is observed in cyanobacterial strain UAB11049 but not shown here due to the genome sequence incompleteness. The impact of differing BGC gene composition on the biosynthesis of tolyporphins products by the strains identified herein requires further investigation.

Overall, the similarities among the predicted BGCs include the following: (i) all of the BGCs contain the unusual cyanobacterial clustering of multiple *hem* genes, including *hemABCEF*, that are adjacent to several *tol*-like genes initially identified in BGC-1 of HT-58-2; (ii) none of these clusters includes *hemD* (UroS); (iii) all *Nostoc* spp. and *Brasilonema* spp. contain two *hemF* genes (the aerobic coproporphyrinogen decarboxylase); (iv) *tolB* (the RfbA orthologue, glucose-1-phosphate thymidylyltransferase) and *tolD* (the glycosyltransferase) are observed in all seven BGCs; and (v) secretory and transport proteins (DevB and DevC families) are encoded by genes near the end of each cluster.

### 3.3. Phylogenetic Relationships among Cyanobacteria and Tolyporphins BGCs Genes

**Figure 5:**
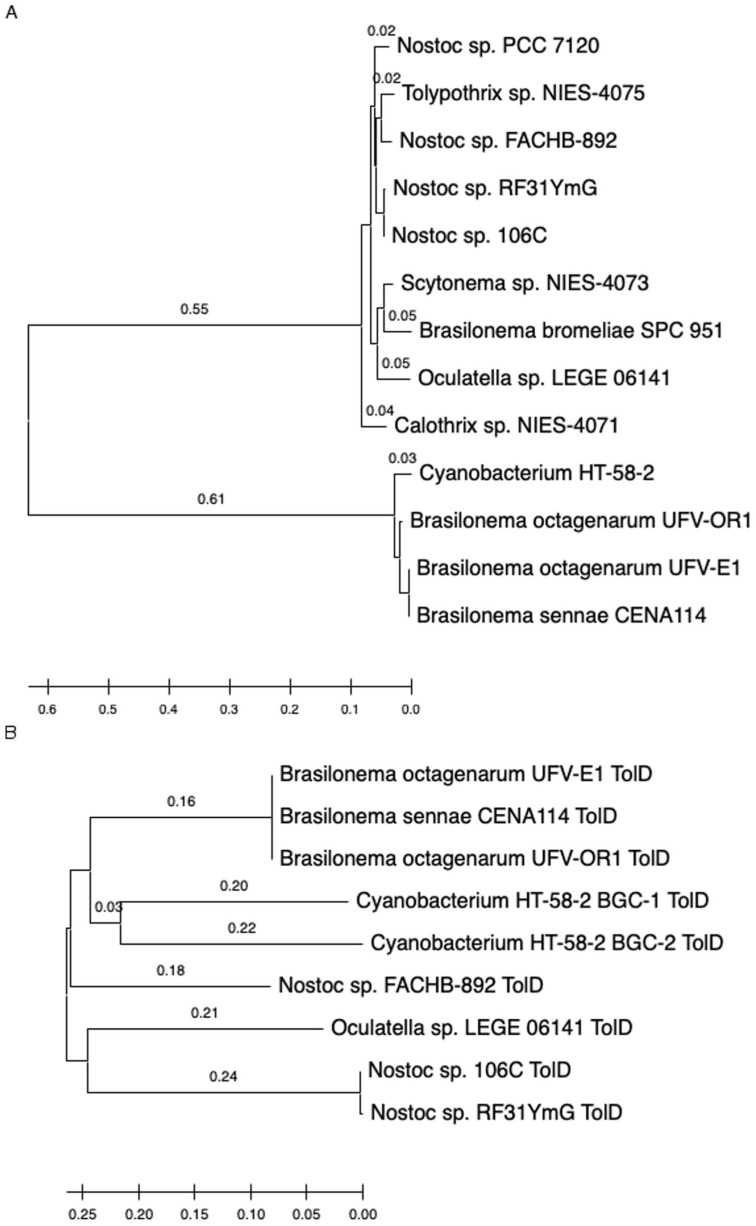
Phylogenetic trees of 16S rRNA (**A**) and TolD (**B**) of cluster-containing cyanobacteria. The trees are drawn to scale with branch unit lengths (above the line) the same as those used to infer the phylogenetic tree. Aligned 16S rRNA sequences were 1027 bases; sum of branch length = 1.49. Aligned TolD proteins were 420 amino acids; sum of branch length = 1.28. Branch length is displayed above each branch line or hidden if shorter than 0.02. All accession numbers are listed in Methods.

To evaluate the relationship between the phylogeny of the cyanobacteria containing related tolyporphins BGCs, the 16S rRNA and protein sequences of one component in the BGCs, termed TolD, were analyzed. The reason for evaluating TolD is that the tolD gene is present in all identified BGCs in the eight cyanobacteria. Figure 5A shows the 16S rRNA phylogenetic tree for cyanobacteria identified with probable tolyporphins BGCs. HT-58-2 clearly shows closer relatedness to three *Brasilonema* strains than to other filamentous cyanobacteria, as previously reported [13]. The TolD phylogenetic tree shows a similar branching pattern as for 16S rRNA (Figure 5B), suggesting the BGCs have the same evolutionary history as the cyanobacteria and were not separately acquired via recent lateral gene transfer. The TolD tree also supports our prior observation [13] that the cyanobacterium HT-58-2 more closely aligns with strains of the genus *Brasilonema* than with those of *Tolypothrix*, as originally described [8].

### 3.4. Examination of Samples from Cyanobacteria with Putative Tolyporphins BGC

For evaluating the potential for tolyporphins production, we obtained and examined three of the cyanobacterial samples that contain a putative tolyporphins BGC: *Nostoc* sp. 106C, *B. octagenarum* UFV-E1, and *Oculatella.* sp. LEGE 06141. The three samples and HT-58-2 were each grown in BG-11 or BG-11o (containing NH_4_HCO_3_, 1.87 mM) for ~4 weeks and then extracted with CH_2_Cl_2_/2-propanol (*v/v* = 1/1) for examination of the presence of tolyporphins. The medium BG-11 contains soluble nitrate whereas BG-11o (containing NH_4_HCO_3_) lacks soluble nitrate; the latter culture medium increases production of tolyporphins in HT-58-2. Regardless, the detection of tolyporphins is challenging due to the presence of chlorophyll as well as the presence of a mixture of up to 18 tolyporphin members. The members can be grouped according to the nature of the chromophore: the dioxobacteriochlorins (tolyporphins A–J and L–O), the oxochlorins (tolyporphins K, Q and R), and a porphyrin (tolyporphin P) [40].

**Figure 6:**
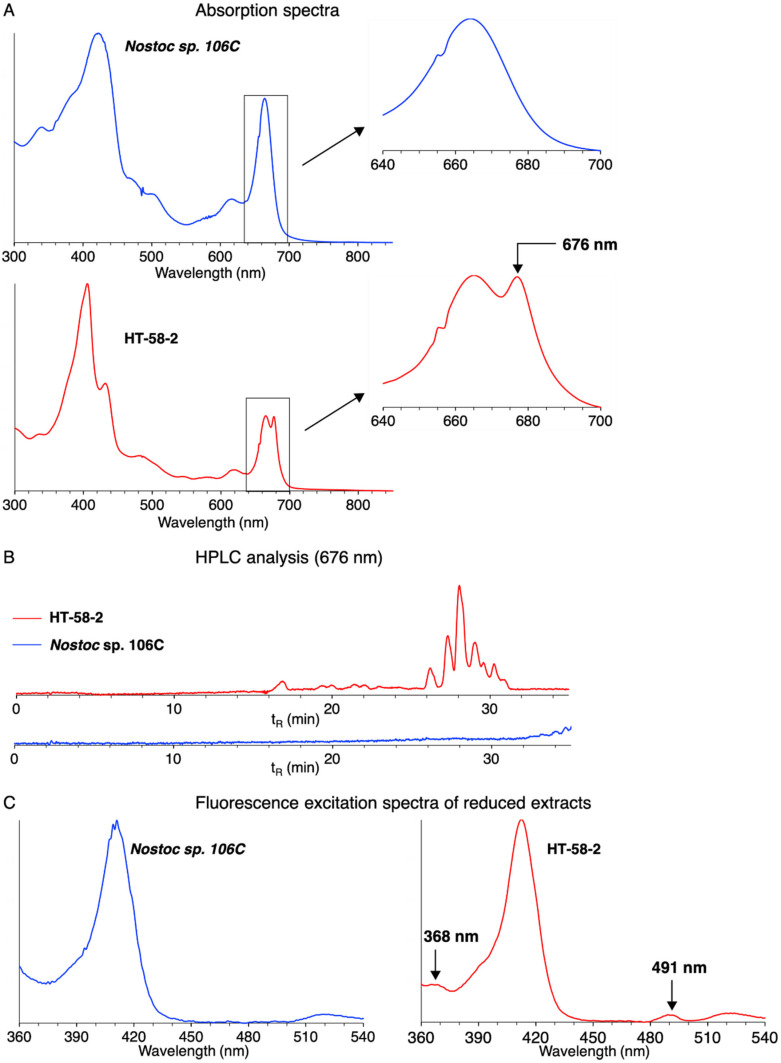
Analytical assays for the presence of tolyporphins in extracts from *Nostoc* sp. 106C (blue lines) and HT-58-2 (red lines) grown in BG11o (containing NH_4_HCO_3_) with continuous white-light illumination for ~4 weeks. Analysis includes absorption spectroscopy (**A**), HPLC with absorption spectroscopic detection (**B**), and an assay using fluorescence excitation spectroscopy (**C**). Results from comparison tests are normalized for intensity (**A**,**C**) or shown at the same level of sensitivity (**B**).

We applied three methods for analysis of culture extracts to detect tolyporphins: absorption spectroscopy [15,40], HPLC with absorption spectroscopic detection [15], and a fluorescence assay [25]. Absorption spectroscopy can be applied directly to lipophilic extracts but is insensitive due to the overlapping signal from chlorophyll; HPLC-absorption spectroscopy is sensitive but requires extensive sample preparation; and the fluorescence assay affords sensitive detection with very limited sample preparation. All three methods were applied to extracts of the three cyanobacterial strains as well as those of HT-58-2. The data for *Nostoc* sp. 106C and HT-58-2 grown in BG-11o (containing NH_4_HCO_3_) are shown in Figure 6.

- The absorption spectral analysis relies on observation of the long-wavelength absorption band (~676 nm) of the dioxobacteriochlorin-type tolyporphins, which constitute the dominant members of the tolyporphins family [15,40]. The spectrum (panel A) of the extract from HT-58-2 indeed shows a peak at 676 nm; such a peak is absent for the extract from *Nostoc* sp. 106C. The peak at 676 nm is a shoulder on the long-wavelength absorption band of chlorophyll *a* (665 nm) [41].
- The HPLC chromatogram with absorption detection at 676 nm (panel B) of the extract from HT-58-2 shows multiple bands with retention time t_R_ ~26–32 min. Such bands are characteristic of the mixture of dioxobacteriochlorin-type tolyporphins. Chlorophyll *a*, which absorbs in the same wavelength region, elutes at longer time [15]. No such bands were observed for the chromatogram of the extract from *Nostoc* sp. 106C.
- The fluorescence assay relies on chemical reduction of the keto auxochromes of chlorophyll *a* and the appropriately substituted tolyporphins followed by fluorescence excitation spectroscopy [25]. The keto groups are present on the dioxobacteriochlorins and oxochlorins, but not the lone porphyrin member, of the tolyporphins family. The fluorescence assay (panel C) for the extract from HT-58-2 showed excitation peaks at 368 and 491 nm (λ_em_ 710 nm) characteristic of dioxobacteriochlorin-type tolyporphins [25], but no such peaks were observed for the extract from *Nostoc* sp. 106C.

Extracts from *B. octagenarum* UFV-E1 and *Oculatella.* sp. LEGE 06141 grown in BG-11o (containing NH_4_HCO_3_) gave similar results in all three analytical methods—no signals characteristic of tolyporphins were observed. Examination of extracts from *Nostoc* sp. 106C, *B. octagenarum* UFV-E1, and *Oculatella.* sp. LEGE 06141 grown in BG-11 by all three analytical methods also gave no signals characteristic of tolyporphins. By contrast, the extract from HT-58-2 affords a positive signal in each analytical method when grown in BG-11o (containing NH_4_HCO_3_). The results are presented in full in the Supplementary Material (Figures S1–S3).

The results lead to the following possible interpretations: (1) The identified BGCs (including in HT-58-2) are not responsible for tolyporphins production. (2) The BGCs are essential for tolyporphins production yet other genes also are required. (3) The identified BGCs suffice for tolyporphins production, but for the newly identified organisms, (i) the appropriate stimuli have not yet been identified, or (ii) tolyporphins are produced but at a level below the limits of detection of, or as a composition that escapes detection by, the three analytical methods employed herein. Further studies are required to probe these possibilities.

## 4. Summary and Outlook

This study has revealed seven new potential producers of tolyporphins based on protein sequences of BGC-1 of cyanobacterium HT-58-2 by the combination of genome mining and comparative genomics analysis. Important questions concern whether tolyporphins are unique to the HT-58-2 culture, how all members of the repertoire of tolyporphins are biosynthesized [42], and the functional roles that tolyporphins play *in vivo*. Focusing on signature components in BGC-1 as targets, such as clustered *hemABCEF* genes, traditional BLASTP searching yielded seven other cyanobacteria with putative tolyporphins BGCs. All of the seven hits are to filamentous cyanobacteria. In what appears to be all cases, the BGC-containing cyanobacteria were isolated from communities associated with plants (*Brasilonema* sp.), in coralloid roots (*Nostoc*. sp. 106C and RF31YmG), or in complex microbial consortia (HT-58-2). Filamentous cyanobacteria living in complex environments might share nutrients or products with other members [43], whereby some of the tolyporphins biosynthetic enzyme candidates may be contributed by other members of the community. The occurrence of the putative tolyporphins BGCs in *Nostoc* and *Brasilonema* clades suggests focus on these genera is warranted to find producers of tolyporphins. Given the apparent yield difference of tolyporphins of HT-58-2 in distinct growth media, the newly identified cyanobacteria require examination under diverse growth conditions including light intensity and periodicity, CO_2_ concentration, nitrogen sources, and carbon sources.

## Figures and Tables

**Table 1:** Information pertaining to cyanobacteria with putative tolyporphins BGCs.

Scheme	Location	Sample Origin	BGC Composition
HT-58-2 BGC-1 ^a^	Pohnpei, Micronesia	Soil	7 *hem* genes, 11 *tol* genes
HT-58-2 BGC-2	Pohnpei, Micronesia	Soil	3 *hem* genes, 7 *tol* genes
*Nostoc* sp.106C	Chiapas, Mexico	Coralloid roots	6 *hem* genes, 7 *tol* genes
*Nostoc* sp. RF31YmG	Chiapas, Mexico	Coralloid roots	6 *hem* genes, 7 *tol* genes
*Nostoc* sp. FACHB-892	Tengger Desert, China	Algal crusts	6 *hem* genes, 6 *tol* genes
*Brasilonema octagenarum* UFV-OR1	Minas Gerais, Brazil	Orchid leaves	8 *hem* genes, 6 *tol* genes
*Brasilonema octagenarum* UFV-E1	Minas Gerais, Brazil	*Eucalyptus grandis* leaves	8 *hem* genes, 6 *tol* genes (assembly gap)
*Brasilonema sennae* CENA114	São Paulo, Brazil	Iron water pipe	8 *hem* genes, 6 *tol* genes
*Oculatella* sp. LEGE 06141	Lagos, Portugal	Green macroalgae	6 *hem* genes, 9 *tol* genes

^a^ HT-58-2 BGC-1 was used as the query template to identify the eight other clusters.

## Data Availability

Data are available from the corresponding authors.

## Supplementary Materials

The following is available online at https://www.mdpi.com/article/10.3390/life11080758/s1, Table S1: Similarity between protein sequences of HT-58-2 BGC-1 and BGC-2 and those from other filamentous cyanobacteria; Figure S1: Normalized absorption spectra of extracts from cultures grown in BG-11 (black traces) or BG-11o (containing NH_4_HCO_3_, red dashed traces); Figure S2: HPLC chromatograms with absorption detection (676 nm) of pigment extracts from cultures grown in (A) BG-11 or (B) BG-11o (containing NH_4_HCO_3_); Figure S3: Normalized fluorescence excitation spectra in methanol of reduced extracts (emission 710 nm) from cultures grown in BG-11 (black lines) or BG-11o (containing NH_4_HCO_3_, blue dashed lines).
