# Supplementary material for: Identification of Putative Biosynthetic Gene Clusters for Tolyporphins in Multiple Filamentous Cyanobacteria
Source: Life (Basel). 2021 Jul 28;11(8):758. doi: 10.3390/life11080758 (PMC8401325; doi:10.3390/life11080758)
Supplement: Supplementary file 1 [file life-11-00758-s001.zip › life-1295455-supplementary.pdf]

Supplementary Material for:

Identification of Putative Biosynthetic Gene Clusters for Tolyporphins in Multiple Filamentous Cyanobacteria

Xiaohe Jin[1], Yunlong Zhang[1], Ran Zhang[1], Kathy-Uyen Nguyen[1],
Jonathan S. Lindsey[1#], and Eric S. Miller[2#]

[1]Department of Chemistry
North Carolina State University
Raleigh, North Carolina 27695-8204, USA

[2]Department of Plant and Microbial Biology
North Carolina State University
Raleigh, North Carolina 27695-7615, USA

#Corresponding Authors:
eric_miller@ncsu.edu
jlindsey@ncsu.edu

Table S1. Similarity between protein sequences of HT-58-2 BGC-1 and BGC-2 and those from other filamentous cyanobacteria.

BGC-1 Protein	BGC-2	*Nostoc* sp. 106C (% identity)	*Nostoc* sp. RF31YmG	*Nostoc* sp. FACHB-892	*B. octagenarum* UFV-E1	*B. sennae* CENA114	*B. octagenarum* UFV-OR1	*Oculatella* sp. LEGE 06141
TolA	N/A	OUL31076 (86.9%)	OUL25679 （86.91%）	N/A	QDL13734 (86.15%)	QDL07372 (86.15%)	NMF65690 (86.15%)	MBE9181909 (46.08%)<br>MBE9181917 (81.62%)
TolB	ARV58419 (82.45%)	OUL31075 (85.67%)	OUL25678 （85.35%）	MBD2726604 (85.67%)	QDL13733 (86.31%)	QDL07371 (86.31%)	NMF65689 (86.31%)	MBE9181916 (82.80%)
TolC	ARV59273 (91.64%)<br>ARV59272 (95.15%)	N/A	N/A	N/A	N/A	N/A	N/A	MBE9181926 (52.03%)
TolD	ARV59277 (65.65%)	OUL31080 (59.85%)	OUL25682 (59.61%)	MBD2726601 (66.03%)	QDL13739 (70.52%)	QDL07377 (70.52%)	NMF65696 (70.52%)	MBE9181920 (60.44%)<br>MBE9181921 (61.52%)
TolE	N/A	N/A	N/A	MBD2726603 (66.06%)	N/A	N/A	N/A	MBE9181917 (22.89%)
TolF	N/A	N/A	N/A	N/A	N/A	N/A	N/A	N/A
TolG	ARV62985 (52.67%)	OUL31082 (74.07%)	OUL25684 (74.29%)	MBD2726588 (27.74%)	QDL13738 (78.30%)	QDL07376 (78.30%)	NMF65695 (78.30%)	MBE9181922 (53.48%)
TolH	ARV62985 (51.22%)	OUL31079 (70.15%)	OUL25681 (70.37%)	MBD2726588 (30.79%)	QDL13738 (68.33%)	QDL07376 (68.33%)	NMF65695 (68.33%)	MBE9181922 (54.50%)

TolI	ARV59271 (58.42%)	OUL31085 (81.77%)	OUL25687 (81.07%)	MBD2726596 (61.69%)	QDL18491 (80.58%)	QDL12115 (80.58%)	NMF61862* (80.58%)	MBE9181928 (59.02%)
TolJ	WP_08753 9995.1 (60.48%)	OUL31084 (59.27%)	OUL25686 (58.84%)	MBD2726597 (56.96%)	QDL13742 (59.01%)	QDL07380 (59.01%)	NMF61861* (59.01%)	MBE9181927 (57.21%)
TolK	N/A	N/A	N/A	N/A	N/A	N/A	N/A	N/A
DUF3102	ARV59266 (51.63%)	N/A	N/A	N/A	N/A	N/A	N/A	N/A
DevB	ARV59268 (73.26%)	OUL31070 (75.13%)	OUL25673 (75.63%)	MBD2726609 (71.43%)	QDL13728 (73.43%)	QDL07366 (73.43%)	NMF65684 (73.43%)	MBE9181911 (66.75%)
DevC	ARV59267 (75.84%)	OUL31069(71.72%)	OUL25672 (72.24%)	MBD2726610 (70.95%)	QDL13727 (74.81%)	QDL07365 (74.81%)	NMF65683 (74.55%)	MBE9181910 (72.49%)

* Denotes proteins similar to TolI and TolJ from *B. octagenarum* UFV-OR1, where the genes are clustered in another contig.

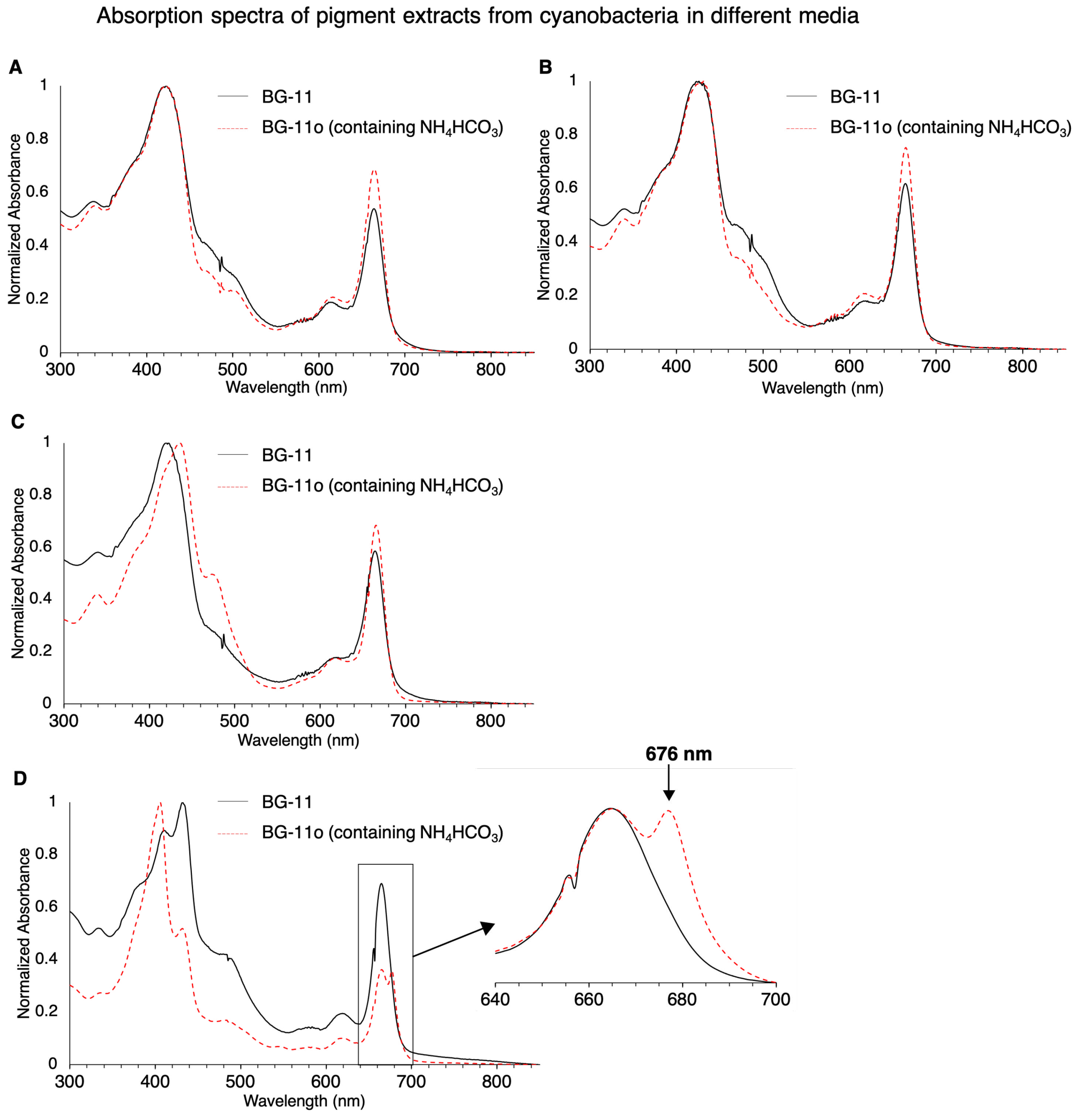

Figure S1. Normalized absorption spectra of extracts from cultures grown in BG-11 (black traces) or BG-11o (containing NH_4HCO_3, red dashed traces). Cultures were grown for ~4 weeks with continuous white-light illumination prior to extraction. (A) *Nostoc* sp. 106C, (B) *Brasilonema octagenarum* UFV-E1, (C) *Oculatella* sp. LEGE 06141, and (D) HT-58-2. The peak at 676 nm is due to dioxobacteriochlorin-type tolyporphins (shown in panel D expansion). The small glitch in absorption at ~656 nm is a characteristic artefact of the diode-array absorption spectrometer.

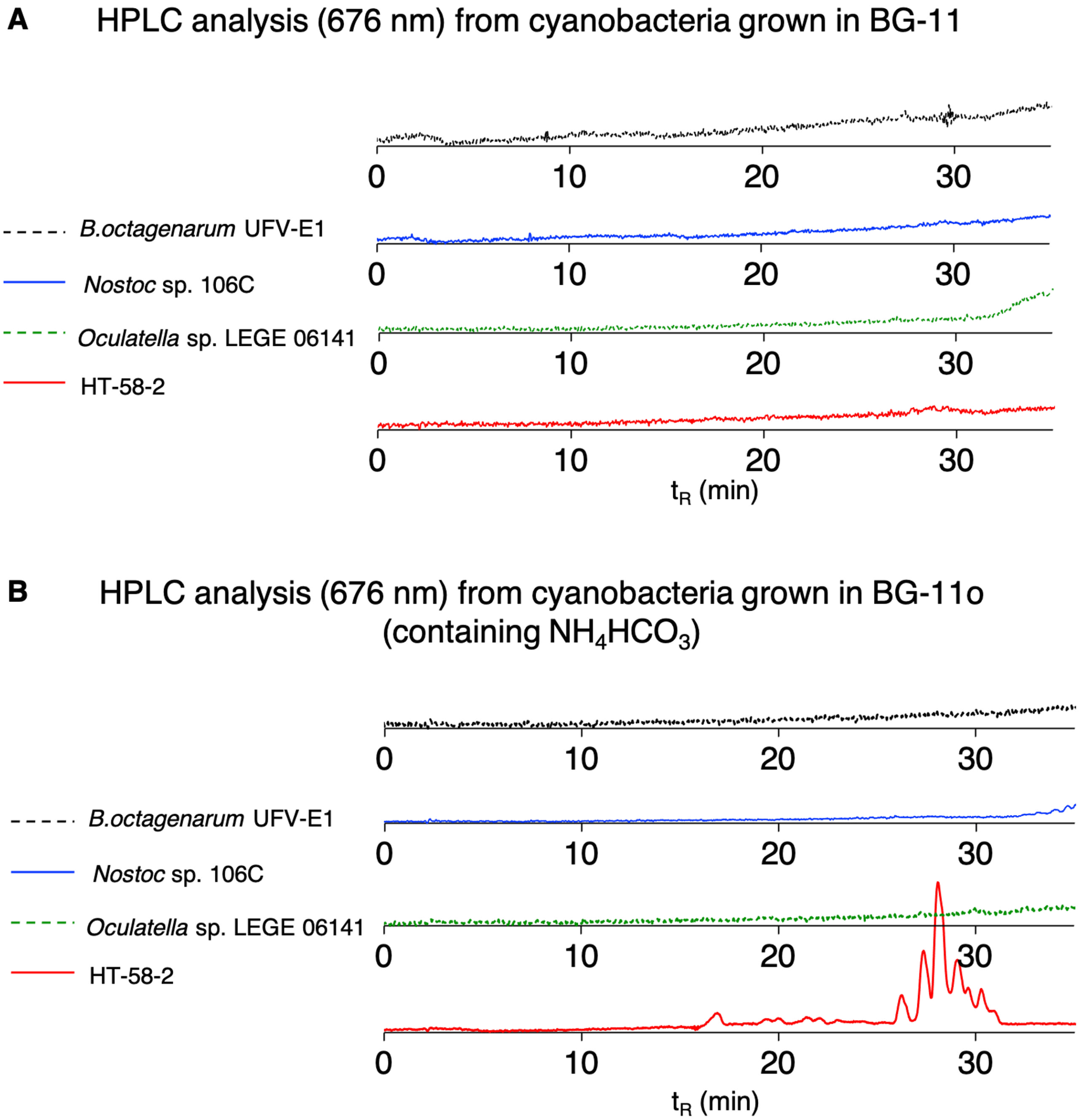

Figure S2. HPLC chromatograms with absorption detection (676 nm) of pigment extracts from cultures grown in (A) BG-11 or (B) BG-11o (containing NH_4HCO_3). Cultures were grown for ~4 weeks with continuous white-light illumination prior to extraction. Chromatograms were obtained for *Brasilonema octagenarum* UFV-E1, *Nostoc* sp. 106C, *Oculatella* sp. LEGE 06141, HT-58-2. Bands of tolyporphins appear at t_R ~26–30 min.

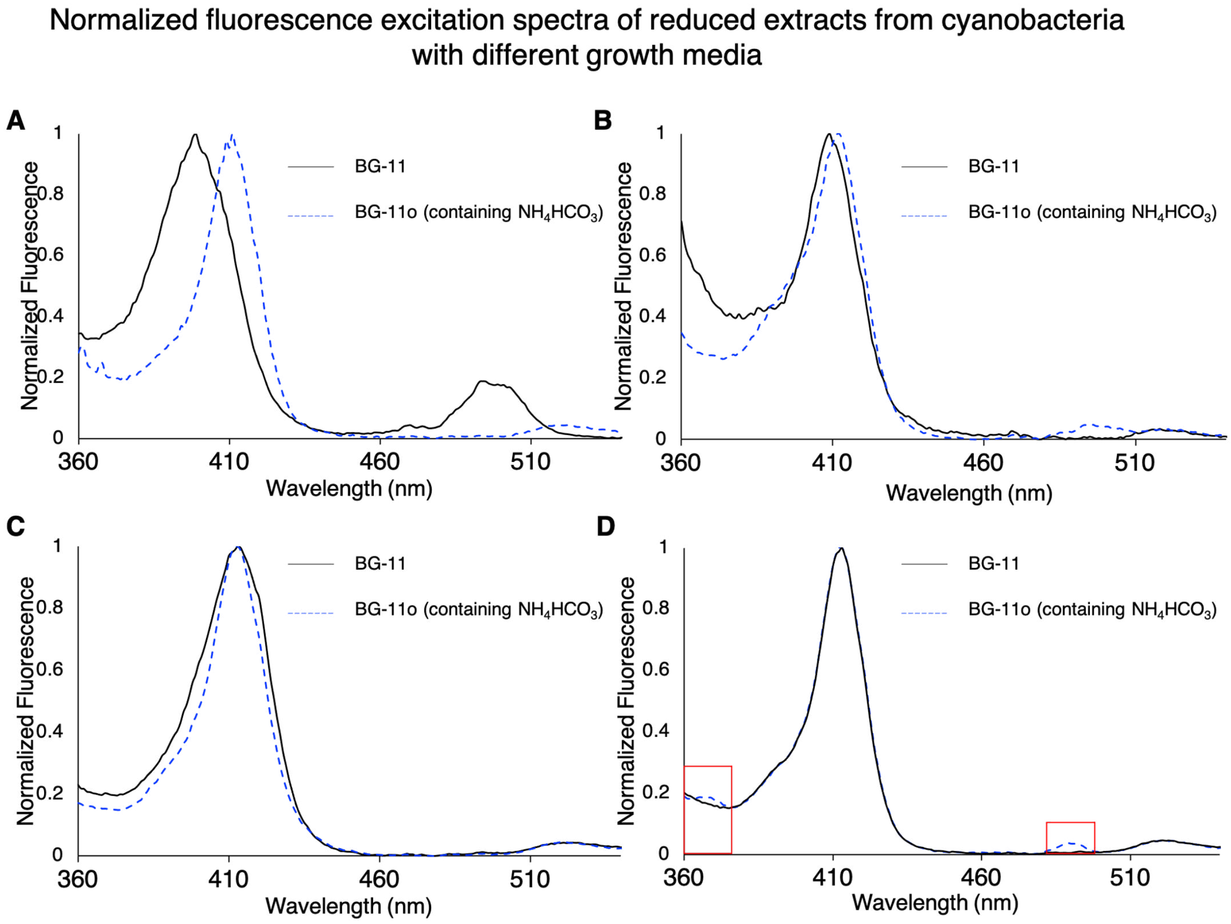

Figure S3. Normalized fluorescence excitation spectra in methanol of reduced extracts (emission 710 nm) from cultures grown in BG-11 (black lines) or BG-11o (containing NH_4HCO_3, blue dashed lines). Cultures were grown for ~4 weeks with continuous white-light illumination prior to extraction. (A) *Nostoc* sp. 106C, (B) *Brasilonema octagenarum* UFV-E1, (C) *Oculatella* sp. LEGE 06141, and (D) HT-58-2. Red boxes highlight the peaks at 368 and 491 nm characteristic of dioxobacteriochlorin-type tolyporphins.
